# The NUDIX hydrolase NUDT5 influences purine nucleotide metabolism and thiopurine pharmacology

**DOI:** 10.1172/JCI194434

**Published:** 2025-07-15

**Authors:** Leo Kager, Kaan Boztug

**Affiliations:** 1 St. Anna Children’s Hospital, Vienna, Austria.; 2 Medical University of Vienna, Department of Pediatrics and Adolescent Medicine, Vienna, Austria.; 3 St. Anna Children’s Cancer Research Institute, Vienna, Austria.; 4 Research Center for Molecular Medicine (CeMM) of the Austrian Academy of Sciences, Vienna, Austria.

## Abstract

Purine nucleotides are critical for nucleic acid synthesis, signaling, and cellular metabolism. Thiopurines (TPs), including 6-mercaptopurine and 6-thioguanine, are cornerstone agents for the treatment of acute lymphoblastic leukemia (ALL). TP efficacy and cytotoxicity depend on the metabolism and intracellular activation of TPs, a process influenced by pharmacogenes such as thiopurine-S methyltransferase (*TPMT*) and NUDIX (nucleoside diphosphates linked to moiety-X) hydrolase 15 (*NUDT15*). In this issue of the *JCI*, Maillard et al. identified *NUDT5* as a determinant of TP pharmacology. They demonstrated that loss of *NUDT5* conferred TP resistance by impairing drug activation and DNA damage responses. Metabolomics studies by Maillard and others revealed that NUDT5 may regulate the balance between the de novo purine synthesis and salvage pathways. Clinically, *NUDT5* expression variants were associated with altered TP tolerance. These findings position *NUDT5* as a key modulator of nucleotide metabolism and TP efficacy, with potential implications for pharmacogenomics-guided therapy optimization in ALL.

## Purine nucleotide metabolism, antimetabolites, and intracellular thiopurine pharmacology

Cells need a constant supply of purine nucleotides (PNs), as PNs play a crucial role in cell signaling, energy transport, metabolism, and DNA and RNA synthesis (1). The availability of cellular PNs depends on the activity of two pathways: the metabolically costly de novo purine synthesis (DNPS) pathway and the purine salvage (PS) pathway; the former consumes approximately six times more ATP molecules per molecule of synthesized purine than the latter (2). The relative contributions of DNPS and PS to cellular PN pools vary according to the cell lineage, extracellular microenvironment, and metabolic state (1).

Rapidly proliferating cells, like cancer cells, require large amounts of PNs. Therefore, pathways that can generate a supraphysiological abundance of intracellular PNs have become attractive anticancer drug targets. The concept of targeted antimetabolite drug therapy for cancer was developed more than 70 years ago. Nobel laureates Gertrude Elion and George Hitchins rationally designed the antineoplastic drugs 6-mercaptopurine (MP) and 6-thioguanine (TG) as thio-substituted purine analogs (thiopurines [TPs]) of hypoxanthine and guanine to target PN metabolism (3). The combination of the antifolate aminopterin, later replaced by methotrexate (MTX), MP, and steroids led to one of the first treatments to induce prolonged, temporary remissions in children with acute lymphoblastic leukemia (ALL) (3). These drugs are still essential elements in the successful treatment of children, adolescents, and adults with ALL (4).

Among TPs, MP and TG are used as antineoplastic agents, whereas azathioprine, a prodrug of MP, is used for immunosuppressive indications (5). This Commentary will focus on TPs in the context of ALL therapy. To exert their antileukemic effects, MP and TG undergo extensive cellular metabolism, which can be influenced by variants in genes associated with the process (6). Activation reactions occur within the PS pathway to form cytotoxic active thioguanine nucleotides (TGNs) (including mono-, di-, and triphosphates [TGMP, TGDP, and TGTP]). TGTPs are incorporated in competition with natural guanine into DNA (as DNA-TG), triggering mismatch repair via MutS homolog 6 (MSH6), DNA strand breaks, and apoptosis (5–7). Of note, higher levels of DNA-TGs in blood leukocytes in vivo during maintenance therapy have been associated with a reduced relapse hazard in children treated for ALL (8). Consequently, the TP-enhanced ALL maintenance (TEAM) strategy, which includes addition of TG to the MP and MTX backbone to enhance DNA-TGs, is currently being investigated in the European ALLTogether1 trial (7).

The anabolic reaction to form cytotoxic TGNs from TPs starts with hypoxanthine-guanine phosphoribosyl transferase 1 (HPRT1), which uses phosphoribosyl pyrophosphate (PRPP) as a cosubstrate to form thioinosine monophosphate (TIMP) or TGMP from MP or TG, respectively. TIMP is a substrate for inosine monophosphate dehydrogenase, which converts TIMP to thioxanthine monophosphate (TXMP). TXMP is then converted to TGMP via guanosine monophosphate synthetase. Catabolism includes methylation of MP, TG, TIMP, and TGMP via thiopurine-S methyltransferase (TPMT), or dephosphorylation of TGNs by NUDIX (nucleoside diphosphates linked to moiety-X) hydrolase 15 (NUDT15) (6, 7).

## Thiopurine pharmacogenomics and acute lymphoblastic leukemia

TPs are an essential component of current ALL treatment protocols, and with modern therapies, more than 90% of children with ALL can be cured (9). Like other anticancer agents, TPs have narrow therapeutic indices. In certain patients, severe myelosuppression can lead to life-threatening infections and drug discontinuation, compromising the efficacy of ALL therapy (10). On the other hand, some leukemia clones, such as very early or early CNS relapse in *TCF3:PBX1*-positive ALL, can reemerge during or soon after maintenance therapy (11, 12). These outcomes suggest that some patients harbor an inherited or acquired resistance to TPs and MTX at prescribed doses. Pharmacogenomic investigations can help to establish mechanistic models to explain why some patients with ALL experience severe TP toxicity, whereas others have ALL blasts that are resistant to TP therapy. Such models can deliver information to guide TP dosing and improve TP therapy. Two important pharmacogenes in the context of TP adverse drug reactions (ADRs) are *TPMT* and *NUDT15*. These genes encode proteins that prevent excessive generation of cellular cytotoxic TGN pools through methylation and dephosphorylation reactions by TPMT and NUDT15, respectively (10). Pathogenic germline loss-of-function variants in either of these genes have been associated with TP toxicity, which accounts for approximately 45% of interpatient variability (10). The Clinical Pharmacogenetics Implementation Consortium (CPIC) provides recommendations of *TPMT/NUDT15* genotype–guided dose adjustments to account for these differences (13).

Pathogenic somatic variants in leukemia blasts from patients with early ALL relapse and associated with resistance to TPs have also been identified in 5′-nucleotidase, cytosolic II (encoded by *NT5C2*), which inactivates TIMPs and TGMPs; phosphoribosyl pyrophosphate synthetase 1 (*PRPS1*), which inhibits the anabolic enzyme HPRT1; and *MSH6*, which influences DNA mismatch repair (14–17). In this issue of the *JCI*, Maillard et al. identified an additional molecule that influences cellular TP pharmacology and pharmacogenomics in ALL — NUDIX hydrolase 5 (NUDT5) (18).

## NUDT5 and the cellular pharmacology of TPs

NUDT15 belongs to a family of 22 hydrolases that dephosphorylate a wide variety of nucleotides, so Maillard et al. hypothesized that other NUDIX members may also affect TP pharmacology (18, 19). The authors first performed a NUDIX-targeted CRISPR/Cas9 screen in two B-ALL cell lines, NALM6 and 697. After 7 days of TG exposure, sgRNAs targeting *NUDT15* were substantially depleted, as expected. Interestingly, sgRNAs targeting *NUDT5* were notably enriched in surviving B-ALL cells. This provided evidence that NUDT5 deficiency drives resistance to TGs. This result agrees with findings from a genome-wide study in the B-ALL cell line REH, in which *NUDT5* was identified as one of the top TP resistance genes (20), and from another high-throughput screen that identified a role of *NUDT5* in TG resistance (21). Next, Maillard et al. showed that *NUDT5* deletion (*NUDT5*^KO^) in NALM-6 and 697 cells did not alter B-ALL cell proliferation. Strikingly, exposure to increasing doses of TG and MP were highly cytotoxic in a dose-dependent manner for NALM6 and 697 B-ALL cells, but had only minimal cytotoxic effects in *NUDT5*^KO^ NALM6 and 697 cells. Reexpression of *NUDT5* in *NUDT5*^KO^ NALM6 cells largely restored TP sensitivity. In addition, they showed that NUDT5 depletion abolished the activation of the DNA damage response pathway after MP treatment.

To provide a mechanistic model of how NUDT5 might affect the cellular pharmacology of TPs, Maillard et al. measured TP-related cytosolic and nucleic metabolites in NALM6 and 697 *NUDT5*^KO^ cells and performed metabolomics analyses and stable isotope tracing assays in NALM6 cells (*NUTD5*^WT^ vs. *NUDT5*^KO^). They found NUDT5 was essential for the activation of TPs to exert their cytotoxic function. Their data suggested that loss of NUDT5 impaired the function of HPRT1, thereby limiting its ability to convert TPs into active metabolites and cause cytotoxic effects. In their model, DNA damage was proposed as the main cause of TP-induced cell death (18).

These findings have even greater importance in light of two other recent studies that also support the role of NUDT5 in TP pharmacology. In one study, Strefeler et al. used uridine salvage and CRISPR/Cas9 screening to identify regulators of de novo pyrimidine synthesis and identified *NUDT5* among the top hits (22). In addition, they found that *NUDT5*^KO^ cells were consistently more resistant to nucleobase analogs that rely on the PRPP amidotransferase (PPAT) such as TPs and 5-fluorouracil (FU) They also performed expanded targeted metabolomics analyses of *NUDT5*^KO^ myelogenous leukemia K562 cells. Their data showed that NUDT5 was an inhibitory binding partner to PPAT, thereby depleting endogenous PRPP pools and impairing nucleobase analog metabolism (22). A second study by Wu et al. (23) corroborated that NUDT5 interacted with PPAT, the rate-limiting enzyme in DNPS that generates phosphoribosylamine. In a series of experiments, they identified NUDT5 as a regulator that governed the balance between DNPS and purine salvage pathways (23). These findings underscore the effect of NUDT5 on nucleotide metabolism and TP efficacy.

## *NUDT5* and TP pharmacogenomics

To evaluate the clinical relevance of *NUDT5* in TP toxicity, Maillard et al. also retrospectively analyzed germline DNA in 582 children with ALL who had been enrolled in the Children’s Oncology Group clinical trial ALLL03N1 (18). They found that patients who were homozygous at the rs55713253 locus had increased NUDT5 expression and required a MP dose reduction (18). Given their in vitro evidence that loss of NUDT5 led to TP resistance in ALL cells, it seems likely that loss-of-function somatic *NUDT5* variants in ALL blast cells in vivo would compromise TP efficacy. In contrast, gain-of-function *NUDT5* germline variants may result in increased TP toxicity, but both of these conclusions require further confirmation.

## Conclusions

These results suggest that NUDT5 plays an important role in nucleotide metabolism to balance DNPS and purine salvage and, consequently, affect TP pharmacology and pharmacogenomics. Many other variables, such as purine availability in vivo, can also influence TP pharmacological effects. For example, Tran et al. have recently shown that the metabolic routes for PN acquisition can vary among tissues and cancers and that dietary nucleotide supplementation can accelerate tumor growth in certain xenograft models (2). Moreover, studies by Zaza et al. provide evidence of differences in DNPS among major ALL subtypes, which may influence TP pharmacodynamics (24). Additionally, the metabolic state and microenvironment within a given tissue are determinants that deserve consideration (1). The findings from Maillard et al. open exciting directions of study to elucidate the underlying mechanism of NUDT5 in PN metabolism and explore how *NUDT5* variants fit into the framework of known influencers of TP pharmacology.

In summary, the identification of NUDT5 as a determinant of nucleotide and TP metabolism presents an important step forward to better understand cellular TP pharmacology. Ultimately, the application of these findings to clinical practice has the potential to further fine-tune TP therapy and improve outcomes for patients with ALL.

## Version 1 07/15/2025

Electronic publication
